# Functional reorganization of the conceptual brain system after deafness in early childhood

**DOI:** 10.1371/journal.pone.0198894

**Published:** 2018-07-05

**Authors:** Natalie M. Trumpp, Markus Kiefer

**Affiliations:** Ulm University, Department of Psychiatry, Ulm, Germany; Universita degli Studi di Udine, ITALY

## Abstract

The neurodevelopmental consequences of deafness on the functional neuroarchitecture of the conceptual system have not been intensively investigated so far. Using functional magnetic resonance imaging (fMRI), we therefore identified brain areas involved in conceptual processing in deaf and hearing participants. Conceptual processing was probed by a pictorial animacy decision task. Furthermore, brain areas sensitive to observing verbal signs and to observing non-verbal visual hand actions were identified in deaf participants. In hearing participants, brain areas responsive to environmental sounds and the observation of visual hand actions were determined. We found a stronger recruitment of superior and middle temporal cortex in deaf compared to hearing participants during animacy decisions. This region, which forms auditory cortex in hearing people according to the sound listening task, was also activated in deaf participants, when they observed sign language, but not when they observed non-verbal hand actions. These results indicate that conceptual processing in deaf people more strongly depends on language representations compared to hearing people. Furthermore, additionally enhanced activation in visual and motor areas of deaf versus hearing participants during animacy decisions and a more frequent report of visual and motor features in the property listing task suggest that the loss of the auditory channel is partially compensated by an increased importance of visual and motor information for constituting object knowledge. Hence, our results indicate that conceptual processing in deaf compared to hearing people is more strongly based on the language system, complemented by an enhanced contribution of the visuo-motor system.

## Introduction

Investigations of early onset deafness allow us to study experience-dependent influences on the neurodevelopment of higher-level cognition functions such as memory and language. In individuals, who became deaf in early childhood before or during language development and did not receive cochlear implants, sign language is typically used instead of spoken language. In this group of deaf people, the neural language system typically used for spoken language in hearing individuals adapts to sign language [1]. Furthermore, due to the lack of the acoustic channel, deaf individuals have to rely on vision, action or touch to gain information about objects and events in the environment [2,3]. These differences in sensory-motor experiences between deaf and hearing people, when interacting with the environment, can result in altered memory traces, which shape the neurodevelopment of higher-level cognition including conceptual representations.

Conceptual representations held in human semantic long-term memory are the basic building blocks of human cognition, because they constitute the meaning of language and thought [4,5,6]. They are an important knowledge base for action planning, problem solving and thinking [7,8,9,10,11]. We asked whether deafness since early childhood induces plasticity in brain circuits underlying conceptual representations and thereby influences the way how the world is conceived in adulthood.

Several earlier studies in deaf people investigated neurodevelopmental changes in sensory processing [12,13]. Other studies focused on the functional-neuroanatomical reorganization of higher-level cognitive functions in deaf people such as working memory [14,15] and different aspects of language processing [16,17]. With regard to sensory processing, studies in deaf individuals demonstrated activation in auditory areas encompassing the superior temporal cortex by visual stimuli [e.g., 12]. In a similar vein, visual working memory tasks (sign language or objects) recruited superior temporal areas in deaf individuals [14]. An analog cross-modal plasticity has also been observed in the visual cortex of the blind [18,19], when they were stimulated with spared sensory modalities [2].

Cross-modal plasticity found at a neural level can be related to an improved behavioral performance for stimuli in the intact sensory modalities: Blind individuals performed better in auditory or tactile tasks compared with seeing individuals [18,20]. Likewise, deaf individuals outperformed hearing individuals in visual motion detection [3].

In addition to cross-modal plasticity during sensory stimulation, neural processing of sign language in deaf individuals has received much interest [1]. This line of research provides insights in the neural organization of a language system, which differs from spoken language with regard to input modality (acoustic vs. visual) and output motor program (speech articulation vs. gesture execution). Despite these differences, sign language activated in deaf signers similar regions in frontal, temporal and parietal cortex, which were also involved in processing spoken language in hearing individuals suggesting a correspondence of the neural representation of the language system in deaf and hearing people [21,22,23]. For instance, phonological processing of signs activated areas in inferior frontal and superior temporal cortex, which were also involved in the phonology of spoken language [16,17]. Most importantly, similar to the observations of cross-modal plasticity described above, sign language activated auditory areas in the superior temporal gyrus and the adjacent temporal plane [21,22,24]. With regard to higher-level cognitive functions, neural circuits underlying working memory have been functionally recognized in deaf individuals and also involve posterior aspects of superior temporal cortex [14]. As posterior superior temporal cortex activation in deaf individuals has been observed for working memory of both signs and non-signs, Cardin and colleagues [14] suggested that this area plays a specific role for working memory functions in deaf individuals, irrespective of the verbal or non-verbal format of the stimuli.

As described above, cross-modal reorganization of auditory areas related to visual stimulation, sign language and higher-level cognition such as working memory are well-documented. However, the neurodevelopmental consequences of deafness on conceptual processing have not been intensively investigated so far.

Although there is a general agreement with regard to the significance of conceptual knowledge for language and higher-level cognition, the functional and neural representation of concepts is matter of a debate. One class of models postulate an amodal system, where sensory or action-related inputs are transformed into a common amodal conceptual representation [25,26,27,28], stored in higher-level heteromodal association cortex. Anterior temporal cortex [29] as well as Wernicke’s area in posterior superior temporal and adjacent parietal cortex [30,31], have been frequently assumed to be the neural basis of an amodal semantic system. According to amodal theories, the loss of the acoustic channel should not induce neuroplastic changes of the conceptual system compared with hearing individuals because conceptual knowledge is assumed to be stored in an amodal format, irrespective of the individual history of sensory-motor experience during concept acquisition.

In contrast to this classical view of conceptual representations, grounded or embodied cognition models propose close links between conceptual memory on the one hand and the sensory and motor systems on the other hand [11,32]. According to this view, concepts are mental entities essentially grounded in modality-specific brain areas representing sensory or action-related information [11,32,33,34,35]. These modality-specific cell assemblies constituting conceptual representations are thought to be formed during concept acquisition depending on the specific individual sensory or motor experience [36,37,38]. In variants of these models, processing in sensory-motor areas, the essential core of the conceptual system, is complemented by processing of verbal associations in language areas within frontal and temporal cortex [11,39,40] as well as by conceptual integration processes in anterior and posterior temporal cortex [11,41]. As experience-dependent plasticity is an essential feature of grounded cognition theories, they predict a neural reorganization of the conceptual system in early onset deaf individuals. The loss of the acoustic channel during concept acquisition should result in a compensatory engagement of the intact visual and motor systems as well as of the language system [42].

The notion of a grounding of conceptual representations in modality-specific brain systems has received empirical support from an increasing number of neuropsychological and brain imaging studies in hearing individuals (for reviews, see [6,11,32] demonstrating that conceptual processing involves sensory and motor areas depending on the relevance of conceptual features [43,44,45,46,47,48].

Furthermore, training studies with novel objects [37,49,50,51] as well as expertise studies with real objects [36,38] indicated an activation of cortical cell assemblies in sensory and motor areas by a conceptual task depending on the specific experience with a given sensory or motor feature of this object class.

However, challenging grounded cognition theories, studies of conceptual processing in blind people indicated an organization of the conceptual brain systems comparable to sighted people [52,53]. Although these findings seem to indicate that the neurodevelopment of the conceptual system does not require sensory experience, it is well possible that in blindness the missing visual channel can be adequately compensated in many instances by touch for gaining information about object form or by audition to infer object motion [2,53]. In deafness, however, missing experience of object sound can less adequately be derived from intact sensory channels, with the exception of object motion or sound-induced vibration [2]. Hence, studying the neural correlates of conceptual processing in deaf people is a highly compelling test for the neuroplasticity of the conceptual system.

The present research had the goal to compare the functional neuroarchitecture of the conceptual system in adult deaf individuals, who received a hearing loss in early childhood but never had cochlear implants, and matched hearing participants using functional magnetic resonance imaging (fMRI). Participants performed an animacy decision task, which probes conceptual processing with non-verbal visual stimuli and, thus, provides a comparable access to conceptual knowledge for both deaf and hearing participants. In addition to the neural correlates of conceptual processing, we determined the semantic content of the concepts presented in the fMRI experiment in deaf and hearing participants using a property listing task after the scanning session. In particular, the generated features of these concepts were evaluated in both participant groups with regard to their reference to the sensory and motor modalities. This allowed us to assess whether auditory deprivation alters the feature composition constituting the semantic content of the concepts under investigation.

In order to assess whether functional cortical reorganization in deafness is based on functional properties of the stimuli (e.g., sign language vs. non-verbal hand actions) or stimulus modality (visual or visuo-motor stimuli in general), we identified in deaf participants brain areas sensitive to observing verbal signs and to observing non-verbal visual hand actions compared to baseline. This issue is important because an earlier study in deaf participants indicated that left superior temporal cortex, which is usually involved in auditory and spoken language processing, is only activated when observers encode hand actions as lexical items of sign language, but not when observers encode the same hand actions as purely visuo-motor stimuli without language relevance [24]. Furthermore, this comparison allows us to determine whether potentially observed activity in superior temporal areas during animacy decision relates to language processing (functional-anatomical overlap only with sign language observation) or to visual or visuo-motor processing in general (functional-anatomical overlap with both sign language and action observation).

In hearing participants, brain areas responsive to environmental sounds and observation of visual hand actions were identified. Contrasting listening to sounds against baseline allowed us to determine auditory brain areas in hearing participants as a comparison to reveal functional reorganization of auditory brain areas in deaf participants. Action observation against baseline reveals the brain systems involved in non-verbal visuo-motor processing as described above. By relating brain activation of the animacy decision task to that of sign language observation, action observation or sound listening in both deaf and hearing participants, respectively, we are able to assess cortical reorganization and to isolate contributions of sensory-motor and language areas to conceptual processing in deaf people.

We expected that early onset deafness induces plastic alterations in the functional-neuroanatomcial organization of the conceptual system: The lack of auditory information might be compensated by an increased importance of verbal associations stored in the language systems. Furthermore, visual and motor channels might be more important for acquiring conceptual object knowledge in deaf people. Compared to hearing people, we therefore expected that animacy decisions in deaf individuals would activate brain areas involved in sign language processing more strongly. Furthermore, based on the assumptions of grounded cognition theories, the higher relevance of visual and motor information for constituting concepts in deaf individuals should result in increased activity in the visual and motor brain systems compared to hearing people.

## Methods

### Participants

Sixteen prelingually deaf participants (deafness at the mean age of 11.38 months, ranging from 0 to 24 months) as well as two participants, who became deaf at the age of three and five years, respectively, participated in this study (8 females). Their mean age was 43.7, ranging from 33 to 66 years. Except for two, all deaf participants were right-handed (according to [54]). All of them had hearing parents. Deafness was mostly caused by meningitis or by unknown factors, rarely by otitis media, oxygen deficiency or pertussis. Importantly, none of our deaf participants had ever had received cochlear implants. All deaf participants reported that German sign language (GSL) is their native language. They attended residential schools for deaf, where they used GSL as their primary language for communication. GSL was also the primary language in sports and other leisure activities according to self-reports. Deaf participants learned spoken language at different times in their life (mean age 4.32 years ranging from 0 (meaning with beginning of language acquisition) to 14 years). Deaf participants either completed high school (n = 8), junior high school (n = 8) or advanced technical college (n = 2).

Deaf participants were compared to eighteen healthy hearing German-speaking volunteers (8 females) which were matched in terms of gender, age (mean 41.3 years, ranging from 19 to 68), handedness, educational background (school graduation hearing: high school (n = 6), junior high school (n = 11), advanced technical college (n = 1); years of education: hearing 13, deaf 14.72) and non-verbal intelligence as assessed with subtest 3 of the Performance Test System developed by Horn [55] (LPS-Score hearing 26.72, deaf 25.78). All participants, deaf and hearing, had normal or corrected-to normal visual acuity and were free from neurological or psychiatric disorders. Hearing participants had normal hearing acuity according to self-report. All participants gave written informed consent. The procedures of the study have been approved by the Ethical Committee of Ulm University. All experiments were performed in accordance with relevant guidelines and regulations. Subjects were paid for participation. In all sessions with deaf participants, a sign language interpreter as well as a deaf research assistant were present in order to facilitate communication.

### Stimuli and procedure—Animacy decision task (experiment 1)

Stimuli in the animacy decision task were 120 pictures of living and non-living objects with differential relevance of acoustic features (e.g., living: rooster/ant, non-living: helicopter/compass). Stimuli denoted well-known common objects, which could be referred to by a sign in GSL. The colored objects (8 bit) were inscribed into a grey colored square of 220 × 220 pixels in order to equate their maximal extension. Pictures were presented for 400 ms in randomized fashion (event-related design) intermixed with trials in which just a blank screen was shown (null events), preceded by 500 ms fixation cross. For each stimulus, subjects had to decide whether the object shown on the picture is living or non-living giving their response with a button press within a time window of 1400 ms before the next trial started. The mean intertrial interval (ITI) was 5.6 s varying randomly between 2.8 and 8.4 s. Stimuli were randomly presented within 2 blocks (duration 11 min.) of 60 trials each (plus 15 null events in each block and 4 practice trials before the first block). Thus, trial sequence was different for each participant across and within blocks. In total 611 functional volumes were acquired.

### Stimuli and procedure—Sign language observation (experiment 2, deaf subjects only)

Sign language observation served to determine areas sensitive to sign language in deaf participants. It allowed us to assess whether areas involved in auditory processing in hearing participants were recruited by visual signs in deaf people, thereby exhibiting cross-modal plasticity [12,13]. For that reason, sign language observation was only administered to deaf participants. Deaf participants were visually presented with 20 videos of signs lexicalized in GSL (3440 ms duration). Videos show our deaf research assistant signing those signs, which denote common everyday objects. Stimuli were presented in 4 blocks (block design) with a duration of 24 s each (5 stimuli per block with a mean interstimulus interval (ISI) of 1360 ms randomly varying between 760 and 1960 ms) alternated by fixation baseline blocks of equal duration. Participants were instructed to ether attentively watch the signs or the fixation cross (during the baseline condition), respectively. Sign language observation as well as action observation and sound listening (see below) were passive viewing tasks in order to avoid response-related motor activation. The four sign language blocks were repeated four times. The experimental session began and ended with a fixation baseline block. In total 396 functional volumes were acquired in 13 min.

### Stimuli and procedure—Action observation (experiment 3)

Action observation was used to determine brain areas involved in the processing of non-verbal hand actions in deaf and hearing participants. Specifically in deaf participants, we could assess whether non-verbal hand actions and sign language, which is based on hand actions to express lexical items, involve the same or a different neural substrate. Stimuli were eight action movies (2 s duration) randomly presented twice in each of 3 blocks (block design with a block duration of 110 s, mean ISI of 4250 ms randomly varying between 2500 and 6000 ms). The stimuli were drawn from an earlier study [56] and showed hands performing an action with an unseen object in front of a black background (the object itself was always removed from the video). During video recording, the actor wore black clothing and performed the action in interaction with real objects in order to ensure that the dynamics of the action were correct. Objects were painted black or covered in black cloth. The hands, which performed the action, were subsequently segmented from the unwanted parts of the scene (actor, object and background). As this action observation experiment was adopted from an earlier study [56], the actor was another one as in the sign language observation experiment. The following eight action categories were presented as movies: 1) screwing with a screwdriver, 2) pounding with a hammer, 3) ironing with an electric iron, 4) typing on a computer keyboard, 5) rolling out with a rolling pin, 6) sweeping with a dustpan, 7) stapling with a stapler and 8) carrying a toolbox. The action observation condition alternated with a fixation baseline condition with equal duration. The experimental session began and ended with a fixation baseline block. Deaf as well as hearing participants were instructed to attentively watch the movies or the fixation cross during the baseline condition. In total 385 functional volumes were acquired within 13 minutes.

### Stimuli and procedure—Sound listening (experiment 4, hearing subjects only)

Listening to sounds allowed us to determine auditory brain areas in hearing participants as a comparison to reveal functional reorganization of auditory brain areas in deaf participants. Ten real sounds from animals (e.g. dogbark) and 10 real sounds from artifactual objects (e.g. helicopter) were used as acoustic stimuli. All acoustic stimuli were adopted from an earlier study [44]. They had a mean duration of 1468 ms (varying between 1050 and 1980 ms, including rise and fall time) and were presented binaurally via closed headphones. All sounds were presented in blocks (block design) with a duration of 24 s each (10 stimuli per block with a mean ISI of 932 ms randomly varying between 383 and 1481 ms). Each acoustic stimulation block was preceded and followed by a resting block (baseline) of equal duration in which only a fixation cross was shown. The acoustic stimulation blocks were presented four times in randomized order. Participants’ task was to attentively listen to the acoustic stimuli while keeping their eyes closed during the whole task. Altogether, 204 functional volumes were acquired within 7 minutes. Due to technical problems with the headphones, five participants did not hear any sounds and thus had to be excluded from statistical analyses in this task.

### Stimuli and procedure—Property listing task (experiment 5)

In this last experiment outside the scanner, participants were presented with written lists of names denoting the objects shown as pictures in experiment 1. We used written names instead of pictures in the property listing task so that participants could not infer visual features directly from the picture. Furthermore, due to the perceptual presence of visual object features, pictures could have induced a bias towards listing predominantly visual features so that features from other modalities such as audition, touch or action would have been neglected. Participants were asked to write down properties they spontaneously associate with. There was no time pressure for the responses, in order to account for the potentially poorer writing skills in the deaf participants. As our deaf research assistant found a written response feasible for the deaf participants, we decided to record written responses and not a signed response in deaf participants for three reasons: (i) We wanted to keep response mode and the entire situation comparable to the hearing participants. (ii) Recording a signed response by the sign interpreter or deaf research assistant would involve a social interaction component potentially biasing results. (iii) Video recording of the signed response for later analyses could distract deaf participants from the task. Each property was classified according to its conceptual feature type using a coding scheme: Sensorimotor features were defined as visual, acoustic, motor (self-executed action), motion-related (observing), tactile, olfactory and gustatory properties, which describe the concept (e.g., cow: has brown fur, moos, can be patted, wags its tail, has bristly fur, smells, delivers tasty milk, respectively). Internal states and emotions were defined as properties that reflect internal cognitive processes or emotional evaluations (e.g., cow: is capricious, beautiful, scary). General associations were defined as properties that are only thematically or symbolically related with the concept (e.g., cow: Alps). Categorical terms were defined as superordinate category labels which can be applied to the target concept (e.g., cow: is an animal, a ruminant, female). In order to test the reliability of the coding scheme, two independent judges classified properties of the same set of concepts yielding an inter-rater-reliability of 76.79%. For statistical analysis, we first calculated the relative frequency for each feature type per concept within each subject. (For instance, a participant reported four properties for a specific concept: two visual features, one general association and one a categorical term. Thus, the relative frequency for visual features was 2/4 = 0.5, for general associations and categorical terms 1/4 = 0.25, each.) In a second step, relative frequencies for each feature type were averaged across all subjects.

### FMRI data acquisition and analysis

Magnetic resonance imaging was performed on a 3.0 Tesla MR system (Siemens Allegra, Erlangen, Germany). For the functional scans, a T2*-weighted single-shot gradient-echo EPI sequence (TE = 38 ms, TR = 2000 ms, flip angle = 90°, matrix 64 x 64 pixels, field of view 210 x 210 mm2, voxel size 3.3 x 3.3 x 4.5 mm3) was used. Starting from the bottom of the brain, 30 transversal slices were acquired in interleaved order. Slice orientation was parallel to a line connecting the base of the frontal and the occipital lobes. Image processing and data analyses were performed with SPM8 (Wellcome Department of Imaging Neuroscience) running under Matlab 2009b (MathWorks, Inc., Natick, MA). Visual and acoustic stimuli were delivered through MR-compatible video goggles and head phones, respectively (Resonance Technology, Los Angeles, U.S.A.). Functionality of the video and sound equipment was checked prior to each experimental session. Experimental control and data acquisition was performed by the ERTS software package (Berisoft, Frankfurt, Germany), except for action observation (Presentation, Neurobehavioral Sytems Inc., Albany, USA). Structural images were acquired with T1-weighted MPRAGE sequence (TR = 2300 ms; TE = 3.9 ms; flip angle = 12°; matrix 256 x 256 pixels, FOV = 256 x 256 mm2, voxel size 1 x 1 x 1 mm3).

Functional images were corrected for differences in slice-timing and head motion and spatially realigned to the mean volume of each session. The realigned images from the animacy decision task (exp. 1) were then normalized to standard MNI space (re-sampled voxel size: 2 x 2 x 2 mm3) using DARTEL [57] and smoothed with a 6 mm FWHM isotropic Gaussian kernel. Images from sign language and action observation as well as from sound listening (exp. 2–4) were normalized to MNI reference brain and smoothed with an isotropic Gaussian kernel of 8 mm FWHM. A temporal high-pass filter with cutoff frequency 1/128 Hz was applied in all experiments.

Statistical analyses were performed at two levels: At the first level, single-subject fMRI responses were modeled by a design matrix comprised of the experimental conditions and the six motion parameters from the realignment procedure convolved with the canonical hemodynamic response function. To allow for inferences at the population level, second-level analyses considered the contrast images of all subjects and treated subjects as random effect per group. In the animacy decision task (exp. 1), the first level analysis comprised the four experimental conditions living versus non-living x high versus low acoustic feature relevance for both groups (deaf vs. hearing). Error and practice trials were modeled as regressors of no interest. We conducted a preliminary fMRI analysis for the animacy decision task, which included the factor acoustic feature relevance. This analysis revealed in hearing participants significantly increased brain activation in temporal cortex related to acoustic feature relevance as expected (peak activation at -46–56 20 mm (MNI coordinates), p < 0.001 uncorrected). However, in deaf participants the animacy decision task elicited strong activation in temporal cortex independent of feature relevance as also the final analyses reveals. Due to this pronounced activation pattern in temporal cortex of deaf participants during the animacy decision task, all between-group comparisons as a function of feature type yielded more activation in deaf than in hearing participants, thereby concealing the subtle feature-specific differences in the hearing group. As analyses of reaction times or error rates in the animacy decision task also did not reveal any interaction between the factors acoustic feature relevance and group, we decided to exclude this factor in the final behavioral and fMRI analyses for the sake of conciseness and simplicity. As our main interest concerned the comparison between hearing and deaf participants in general, we thus contrasted the main effect of all conditions in deaf versus hearing subjects in a two-sample t test for second-level analysis. For sign language/action observation and sound listening (exp. 2–4), stimulation blocks (exp. 2: signs; exp. 3: action movies; exp. 4: sounds) were used as experimental condition in the first-level analyses. Resulting contrast images (against baseline) were then subjected to one sample t-tests at the second level. All within-group comparisons (against baseline) were thresholded at a significance level of p < 0.05, corrected for multiple comparisons across the entire brain (family wise error, FWE). For differential effects between groups, effect sizes are naturally smaller than the comparisons against baseline. Therefore, comparisons between groups were thresholded at a more lenient significance level of p < 0.001 uncorrected for multiple comparisons. In addition, we report which cluster and voxel are also significant at a threshold of p < 0.05 FWE-corrected for multiple comparisons. We performed several analyses using activation during sign language/action observation and sound listening as inclusive or exclusive masks, respectively, to determine the functional overlap between tasks. For inclusive masking, we saved results of the three main contrasts watching GSL signs, watching actions and listen to sounds versus baseline as images, respectively, and used them as masks (or templates) in region of interest (ROI) analyses for the task in question (e.g., animacy decision). Hence, in inclusive masking, the masks were comprised of significant voxels for sign language/action observation or sound listening. As a consequence, inclusive masking analyses yielded only suprathreshold voxels for a given task, which also spatially overlap with the mask, i.e. which also show suprathreshold activation for sign language/action observation or sound listening. For exclusive masking, which was performed for sign language and action observation as masks, we saved the results of the main contrast watching signs/actions versus baseline as images and created negative images. These negative images were only comprised of voxels, which were not significant (i.e. not included) for sign language or action observation, respectively. These negative images were then used as masks for the ROI analyses for the task in question.

## Results

We first report the results of the property listing task (originally administered to the participants after the scanning session) before going into detail on the fMRI experiments. In the last section, we describe the functional overlap across tasks using inclusive and exclusive masking analyses.

### Property listing task (experiment 5)

Overall, participants listed about two properties per concept on average. Although the difference was small, hearing participants listed significantly more properties than deaf participants (2.5 vs. 2.0 properties, t(34) = 2.07, p = .046). Despite these slight differences, this observation demonstrates that deaf participants appropriately performed the task. All participants reported properties related to sensorimotor features most frequently followed by general associations and categorical terms or internal states and emotions, respectively (Table 1). Within the sensorimotor features, deaf as well as hearing subjects listed visual features most frequently followed by motor and acoustic features. All other sensorimotor features (motion-related, tactile, olfactory and gustatory) were reported rarely.

**Table 1:** Relative frequency and standard deviation (SD) for each feature type listed in the property listing task for deaf and hearing participants, respectively.

feature	deaf (SD)	hearing (SD)	p
**sensorimotor features**	0.56 (0.13)	0.45 (0.10)	**< 0.001**
visual	0.32 (0.12)	0.26 (0.08)	**< 0.0001**
acoustic	0.04 (0.03)	0.08 (0.04)	**0.03**
motor	0.14 (0.07)	0.06 (0.04)	**< 0.0001**
motion-related	0.04 (0.03)	0.02 (0.02)	0.28
tactile	0.02 (0.02)	0.02 (0.02)	0.83
olfactory	0.00 (0.00)	0.00 (0.00)	1.00
gustatory	0.00 (0.00)	0.00 (0.01)	1.00
**internal states and emotions**	0.04 (0.04)	0.10 (0.10)	0.15
**general associations**	0.15 (0.09)	0.36 (0.09)	**< 0.001**
**categorical terms**	0.13 (0.11)	0.07 (0.07)	0.19

P values result from post-hoc Newman-Keuls tests of the significant interactions between group (deaf vs. hearing) and feature.

In order to identify differences between groups, we first analyzed in a mixed design analysis of variance (ANOVA) with the between-subject factor group (deaf vs. hearing) and the within-subject factor feature type relative frequency of listings of sensorimotor features, internal states and emotions, general associations and categorical terms. This analysis revealed a significant main effect of feature type (F(3,102) = 119.10; p < 0.0001) which is based on significant global differences between all feature types (Newman-Keuls tests all p < 0.001), except for internal states and emotions versus categorical terms (p = 0.26). The analysis also revealed a significant interaction between group and feature type (F(3,102) = 15.64; p < 0.0001): According to Newman-Keuls tests, deaf compared to hearing subjects reported sensorimotor features with a significantly higher relative frequency, but general associations with a significantly lower relative frequency (both p < 0.001). Relative frequency of internal states and emotions and categorical terms were comparable for deaf and hearing participants (both p > 0.1). The second ANOVA comparing the different sensorimotor features in detail also revealed a significant main effect of feature type (F(6,204) = 165.29; p < 0.0001) and a significant interaction between group and feature type (F(6,204) = 6.58; p < 0.0001). The interaction was further explored with Newman-Keuls tests: Deaf participants reported visual and motor features with a higher relative frequency than hearing participants (both p < 0.0001), but acoustic features with a lower relative frequency (p < 0.05). Motion-related, tactile, olfactory and gustatory features were comparable across groups (all p > 0.1).

### Animacy decision task (fMRI experiment 1)

Reaction times (RT) and error rates (ER) in the animacy decision task were subjected to separate mixed design ANOVAs with the between-subject factor group (deaf vs. hearing) and the within-subject factor category (living vs. non-living). Analysis of RT data (mean deaf: 693 ms (SD 174 ms); mean hearing: 634 ms (SD 108 ms)) revealed no significant main effect of group (F(1,34) = 1.49, p = 0.23), but a significant main effect of category (F(1,34) = 17.76, p < 0.001). All participants were significantly faster responding to pictures of living compared to non-living objects (deaf: living 684 ms (SD 171 ms), non-living 702 ms (SD 182 ms); hearing: living 612 ms (SD 104 ms), non-living 656 ms (SD 111 ms)). Thus, general RT performance was similar across groups. Analysis of ER (deaf: living 2.69% (SD 2.37%), non-living 1.11% (SD 1.51%), overall 1.90% (SD 2.11%); hearing: living 2.13% (SD 3.01%), non-living 3.70% (SD 4.80), overall 2.92% (SD 4.03%)) yielded neither a significant main effect of group (F(1,34) = 1.24, p = 0.27) nor of category (F(1,34) < 0.001, p = 1.00), but a significant interaction between both factors (F(1,34) = 9.11, p < 0.005). Post-hoc Newman-Keuls tests, however, revealed no significant differences within or across groups.

**Fig 1:**
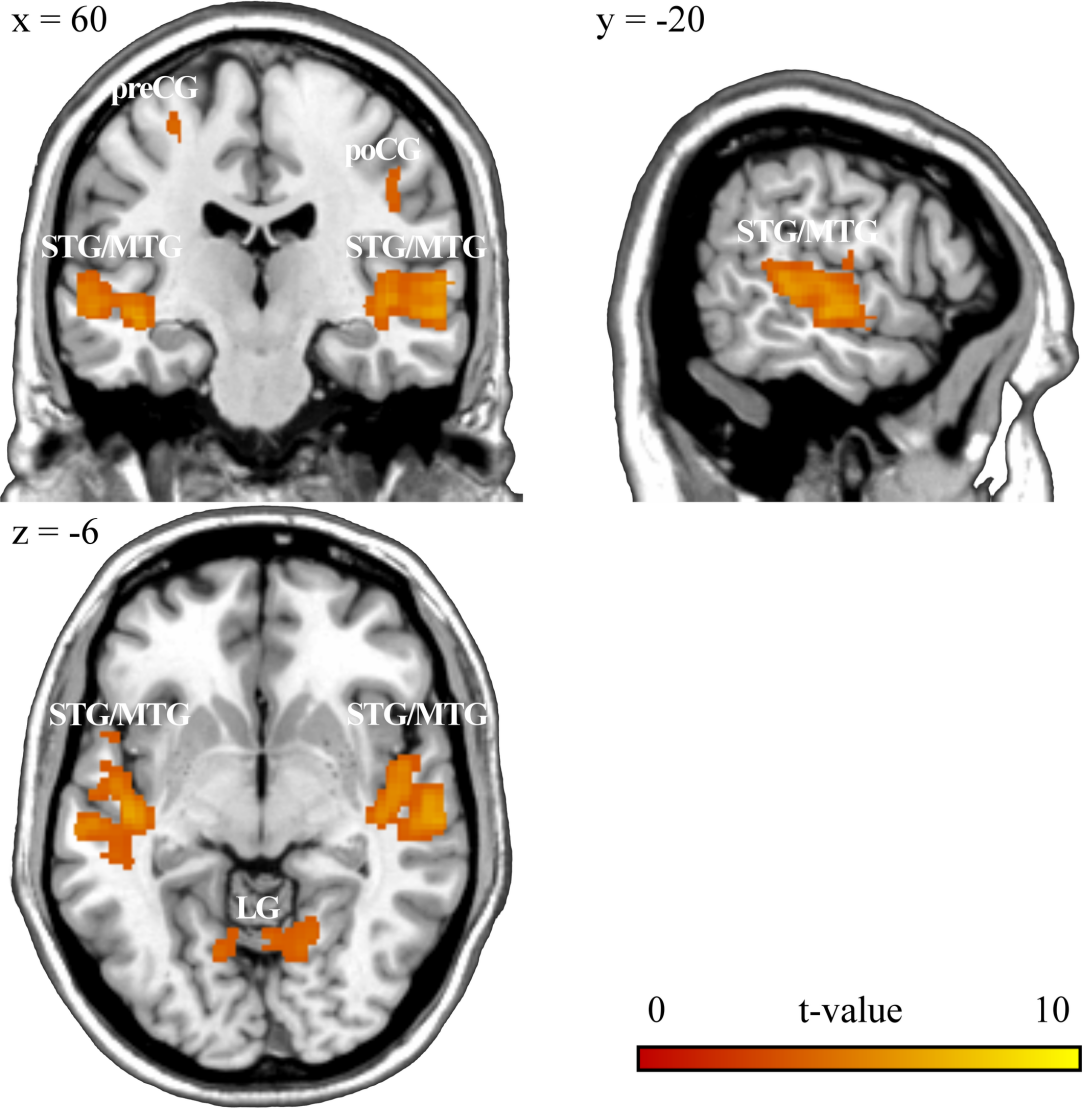
Functional brain activation during the animacy decision task in deaf versus hearing participants (p < 0.001, uncorrected). STG: superior temporal gyrus, MTG: middle temporal gyrus, preCG: precentral gyrus, poCG: postcentral gyrus, LG: lingual gyrus.

Statistical analysis of the fMRI data revealed significantly increased brain activation during the animacy decision task compared to baseline in deaf as well as in hearing subjects in widespread parts of the brain involving temporal, occipital, frontal and parietal cortical regions as well as subcortical structures (thalamus, basal ganglia and hippocampus) and the cerebellum (for a detailed overview, see S1 Table). Our main interest, however, concerned differential effects between deaf and hearing subjects (Fig 1, Table 2): Comparing brain activity of hearing versus deaf participants did not reveal any suprathreshold voxels. However, in deaf versus hearing participants, the MR signal was significantly increased within temporal (bilateral superior/middle temporal gyrus, BA 22/21/48), occipital (bilateral lingual gyrus as well as bilateral calcarine sulcus and left cuneus, BA 18/19), frontal (right precentral gyrus, BA 4/6 and right pars opercularis, BA 44) and parietal brain regions (right postcentral gyrus, BA 3) as well as within vermis and left cerebellum. Increased activation in the superior and middle temporal cluster in deaf compared to hearing participants remained significant at a more restrictive statistical threshold of p < 0.05 FWE-corrected for multiple comparisons. Increased activity in the occipital cluster also remained significant at a threshold of p < 0.05 FWE-corrected. Thus, in deaf participants animacy decision was robustly associated with increased activity in temporal cortex as well as within parts of the visual system (occipital cortex). Activity differences in motor brain regions (frontal and parietal cortex) were also found, but at a more lenient significance level.

**Table 2:** Activation peaks for differential effects between deaf and hearing subjects in the animacy decision task (activation during animacy decision in deaf vs. hearing /hearing vs. deaf participants).

Brain region	BA	MNI coordinates(mm)	T	P_Voxel_ _uncorr_	Cluster size	P_Cluster_ _uncorr_
***deaf vs*. *hearing subjects***						
Middle temporal L	21	-44–18–8	7.14	< 0.0001*	1443	< 0.0001*
Middle temporal L	21	-62–24 2	6.04	< 0.0001*		
Superior temporal L	22	-50–6–8	5.93	< 0.0001*		
Middle temporal R	21	60–20–6	6.44	< 0.0001*	1697	< 0.0001*
Superior temporal R	22	54–32 8	6.29	< 0.0001*		
Superior temporal R	48	50–26 4	6.23	< 0.0001*		
Inferior frontal pars opercularis R	44	54 10 28	4.83	< 0.0001	57	0.033
Lingual R	18	14–66–4	4.68	< 0.0001	220	< 0.0001*
Vermis		4–60–4	4.21	< 0.0001		
Calcarine L	18	-10–70 22	4.63	< 0.0001	723	< 0.0001*
Cuneus L	18	6–72 22	4.56	< 0.0001		
Calcarine R	18	24–62 16	4.54	< 0.0001		
Lingual L	19	-18–46 0	4.56	< 0.0001	85	0.012
Postcentral R	3	52–4 32	4.37	< 0.0001	53	0.039
Precentral R	6	42–8 40	3.84	< 0.0001		
Lingual L	18	-10–72 0	4.22	< 0.0001	88	0.01
Lingual L	18	-16–66–6	4.18	< 0.0001		
Cerebelum L		-8–58–6	3.59	0.001		
Precentral R	4	18–28 56	4.14	< 0.0001	57	0.033
***hearing vs*. *deaf subjects***						
No suprathreshold voxels						

The statistical threshold was set to p < 0.001 uncorrected for the whole brain (* also P_FWE-corrected_). Shown are peak voxels with highest t-values for significant clusters and their local maxima more than 8 mm apart. BA: Brodmann Area, MNI: Montréal Neurological Institute, R: right, L: left. See also S1 Table.

### Activity to sign language in deaf participants (fMRI experiment 2)

**Fig 2:**
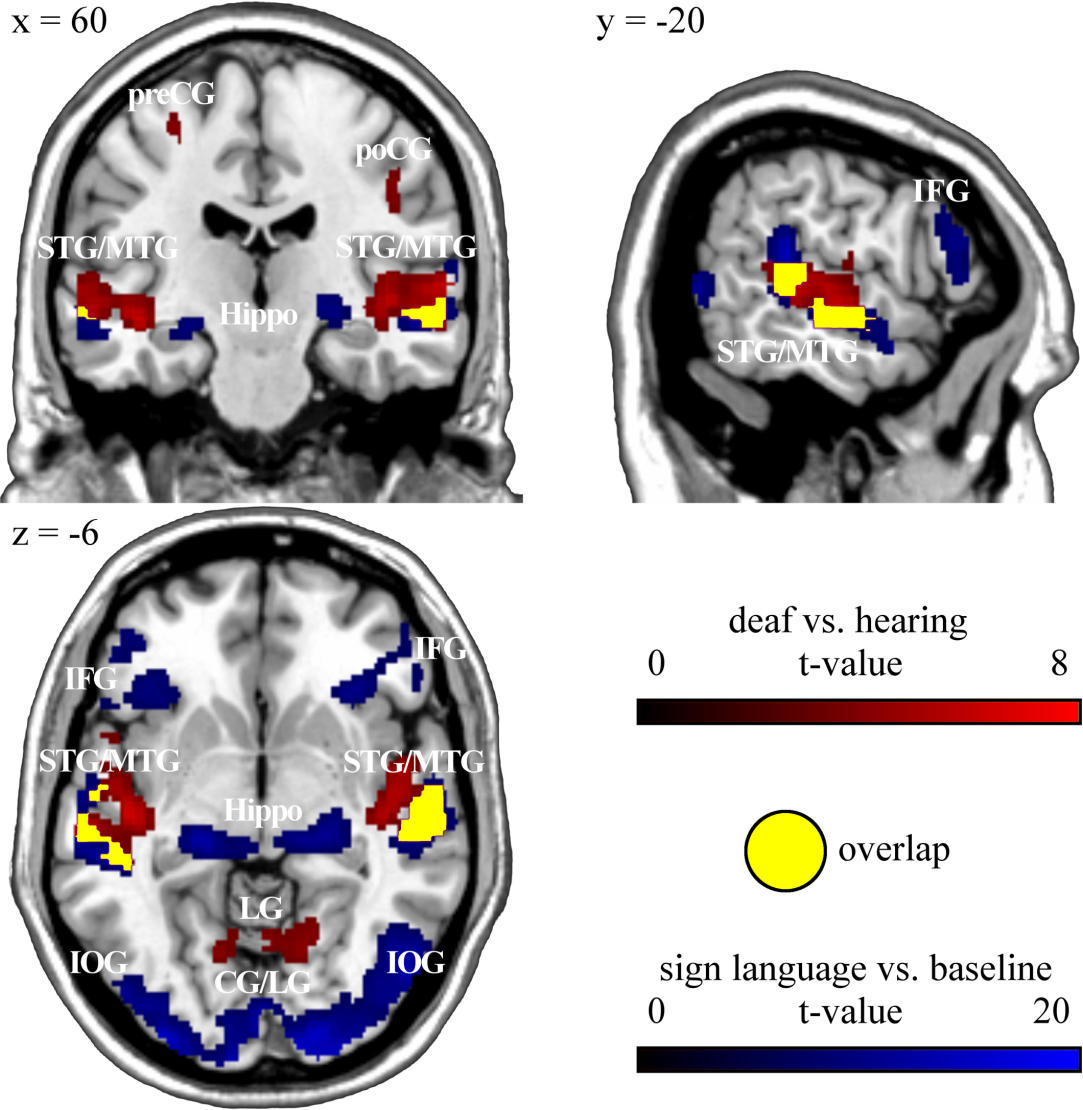
Functional-anatomical overlap between activation during the animacy decision task in deaf versus hearing participants (p < 0.001, uncorrected) and activity to sign language compared to baseline in deaf participants (p < 0.05, FWE corrected). STG: superior temporal gyrus, MTG: middle temporal gyrus, preCG: precentral gyrus, poCG: postcentral gyrus, Hippo: hippocampus, IFG: inferior frontal gyrus, LG: lingual gyrus, CG: calcarine gyrus, IOG: inferior occipital gyrus.

When viewing movies of lexical GSL signs compared to baseline, deaf participants showed a significantly increased MR signal within bilateral temporal (superior/middle temporal gyrus, BA 42/20, 21, 22), bilateral occipital (middle/inferior temporal gyrus, BA 19, 37; superior/middle/inferior occipital gyrus, BA 17, 18, 19; calcarine sulcus, BA 17/18; lingual gyrus, BA 18/19), bilateral frontal (bilateral precentral and right middle frontal gyrus as well as left supplementary motor area, BA 6/8; pars opercularis/triangularis (Broca), BA 44/45) and bilateral parietal brain regions (superior/inferior parietal gyrus, BA 7/40) as well as within left cerebellum, bilateral hippocampus and right amygdala. (Fig 2, Table 3).

**Table 3:** Activation peaks for the three main contrasts watching GSL signs and actions or listen to sounds vs. baseline.

Brain region	BA	MNI coordinates (mm)	T	P_Voxel_ _FWE-corr_	Cluster size	P_Cluster_ _FWE-corr_
***Sign language observation—deaf subjects***						
Superior temporal R	42	64–34 16	20.62	< 0.0001	1393	< 0.0001
Middle temporal R	21	56–22–8	13.51	< 0.0001		
Middle temporal R	21	54 4–18	13.18	< 0.0001		
Middle occipital R	17	24–98 6	19.23	< 0.0001	10122	< 0.0001
*Inferior temporal R*	*19*	*52–72–2*	*17*.*96*	*< 0*.*0001*		
*Calcarine L*	*17*	*2–86–2*	*17*.*32*	*< 0*.*0001*		
*Inferior frontal pars opercularis L*	*44*	*-48 12 28*	*17*.*30*	*< 0*.*0001*		
*Superior occipital L*	*17*	*-12–104 12*	*17*.*14*	*< 0*.*0001*		
*Lingual R*	*18*	*18–96–8*	*16*.*03*	*< 0*.*0001*		
*Middle occipital L*	*18*	*-32–96–6*	*16*.*01*	*< 0*.*0001*		
*Inferior temporal R*	*37*	*54–66–2*	*15*.*93*	*< 0*.*0001*		
*Calcarine R*	*18*	*10–98–4*	*15*.*21*	*< 0*.*0001*		
*Calcarine R*	*17*	*12–100 2*	*14*.*78*	*< 0*.*0001*		
*Middle temporal L*	*37*	*-56–62 2*	*14*.*76*	*< 0*.*0001*		
*Inferior occipital L*	*19*	*-44–72–14*	*14*.*18*	*< 0*.*0001*		
*Middle occipital L*	*19*	*-48–80 0*	*13*.*53*	*< 0*.*0001*		
*Inferior occipital R*	*19*	*44–74–12*	*13*.*07*	*< 0*.*0001*		
*Inferior occipital R*	*18*	*28–96–12*	*13*.*05*	*< 0*.*0001*		
*Middle temporal L*	*21*	*-50 0–20*	*13*.*04*	*< 0*.*0001*		
*Inferior frontal pars triangularis L*	*45*	*-52 36 12*	*12*.*75*	*< 0*.*0001*		
*Cerebellum L*		*-42–58–24*	*12*.*60*	*< 0*.*0001*		
*Middle temporal L*	*20*	*-58–24–12*	*12*.*55*	*< 0*.*0001*		
*Middle temporal L*	*22*	*-58–4–12*	*12*.*28*	*< 0*.*0001*		
*Precentral L*	*6*	*-46 0 56*	*11*.*79*	*< 0*.*0001*		
*Lingual L*	*19*	*-36–88–16*	*11*.*58*	*< 0*.*0001*		
Inferior frontal pars triangularis R	45	52 42 18	15.27	< 0.0001	1208	< 0.0001
Inferior frontal pars triangularis R	48	54 18 22	12.00	< 0.0001		
Inferior frontal pars triangularis R	48	50 22 16	11.06	< 0.0001		
Superior parietal R	7	34–62 58	14.99	< 0.0001	272	< 0.0001
Inferior parietal R	40	40–48 50	9.71	0.002		
Hippocampus R	20	22–26–2	14.29	< 0.0001	425	< 0.0001
Hippocampus R	20	28–14–10	8.53	0.008		
Supplementary motor area L	6	0 10 60	13.38	< 0.0001	573	< 0.0001
Supplementary motor area L	6	-6 2 66	9.10	0.004		
Supplementary motor area L	8	-4 24 52	8.31	0.011		
Hippocampus L	20	-20–26–6	12.98	< 0.0001	332	< 0.0001
Middle frontal R	6	44 0 60	10.58	0.001	380	< 0.0001
Middle frontal R	6	50 6 54	10.39	0.001		
Precentral R	6	48 8 42	9.55	0.002		
Inferior parietal L	40	-40–46 54	9.68	0.002	145	< 0.0001
Hippocampus R	35	16–6 54	9.21	0.003	42	< 0.0001
Amygdala R	34	22 0–18	8.20	0.012		
***Action observation–deaf subjects***						
Middle temporal L	37	-58–62 2	8.69	< 0.0001	537	< 0.0001
Inferior occipital L	19	-44–76–2	6.42	0.003		
Inferior temporal R	37	56–62–2	7.99	< 0.0001	564	< 0.0001
Inferior occipital R	19	48–74–12	5.93	0.012		
Superior temporal L	42	-60–36 22	6.50	0.003	119	< 0.0001
Supramarginal L	2	-64–30 38	5.38	0.045		
Inferior frontal pars opercularis L	44	-48 10 22	5.91	0.012	30	0.006
***Action observation–hearing subjects***						
Middle occipital L	37	-36–68 2	8.12	< 0.0001	710	< 0.0001
Fusiform L	37	-46–56–18	7.17	0.001		
Middle temporal L	37	-52–66 4	6.93	0.001		
Middle temporal R	37	44–68–2	7.28	< 0.0001	525	< 0.0001
Middle occipital R	19	50–78 2	6.72	0.002		
Inferior occipital R	19	46–74–12	6.32	0.004		
Inferior frontal pars opercularis L	44	-52 10 20	5.91	0.012	48	0.002
Inferior frontal pars triangularis L	45	-50 34 6	5.83	0.015	53	0.002
***Sound listening—hearing subjects***						
Middle temporal L	22	-62–18 2	13.00	0.002	190	< 0.0001
Superior temporal L	22	-60–6–4	12.44	0.003		
Middle temporal L	48	-50–22 2	12.05	0.004		
Superior temporal R	21	62–28 2	12.50	0.003	76	< 0.0001
Superior temporal R	21	48–30 4	9.75	0.031		
Superior temporal R	22	60–12 2	10.80	0.014	37	< 0.0001

The statistical threshold was set to p < 0.05, FWE corrected for the whole brain (cluster size ≥ 30 voxel). Shown are peak voxels with highest t-values for significant clusters and their local maxima more than 8 mm apart. For clusters comprising more than 10000 voxels embedded local maxima more than 4 mm apart are listed (italic) in order to give a better overview of the involved brain regions. BA: Brodmann Area, MNI: Montréal Neurological Institute, R: right, L: left.

### Activity to action observation in deaf and hearing participants (fMRI experiment 3)

**Fig 3:**
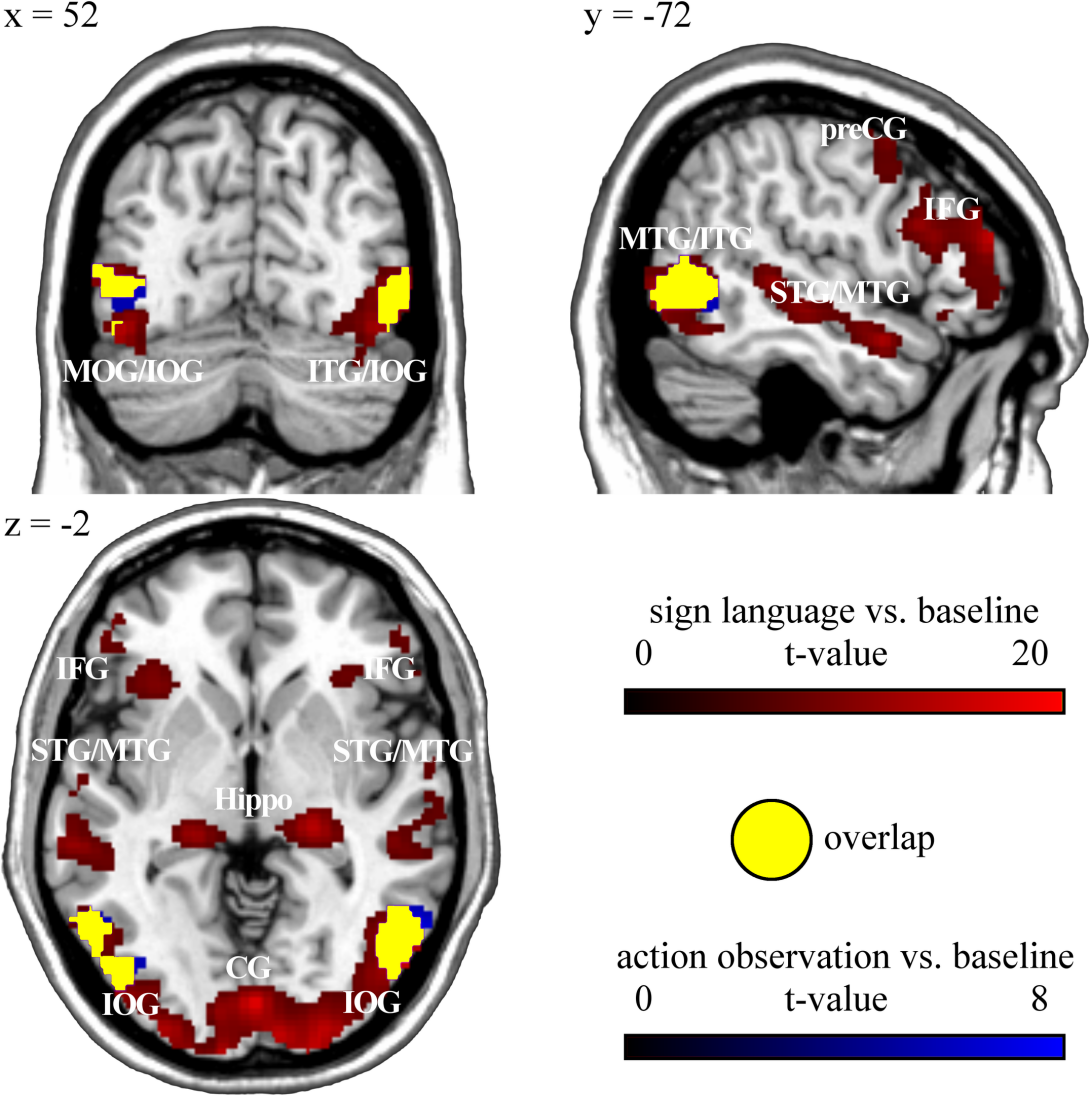
Functional-anatomical overlap between activity to sign language compared to baseline in deaf participants (p < 0.05, FWE corrected) and activity to action observation compared to baseline in deaf participants (p < 0.05, FWE corrected). MOG: middle occipital gyrus, IOG: inferior occipital gyrus, ITG: inferior temporal gyrus, STG: superior temporal gyrus, MTG: middle temporal gyrus, preCG: precentral gyrus, IFG: inferior frontal gyrus, Hippo: hippocampus, CG: calcarine gyrus.

Deaf and hearing subjects showed significantly increased brain activation during observation of (non-verbal) hand actions compared to baseline in similar brain regions encompassing bilateral fusiform gyrus (inferior/middle temporal and middle occipital gyrus, BA 37), inferior/middle occipital gyrus (BA 19; deaf bilateral, hearing only left-hemispheric), left inferior frontal gyrus pars opercularis/triangularis (BA 44/45). Deaf subjects additionally activated left superior temporal and left supramarginal gyrus (BA 42/2). However, contrasting brain activation during action observation of deaf versus hearing participants and vice versa (p < 0.001 uncorrected) did not reveal any suprathreshold voxels (Fig 3, Table 3).

### Activity to sounds in hearing participants (fMRI experiment 4)

**Fig 4:**
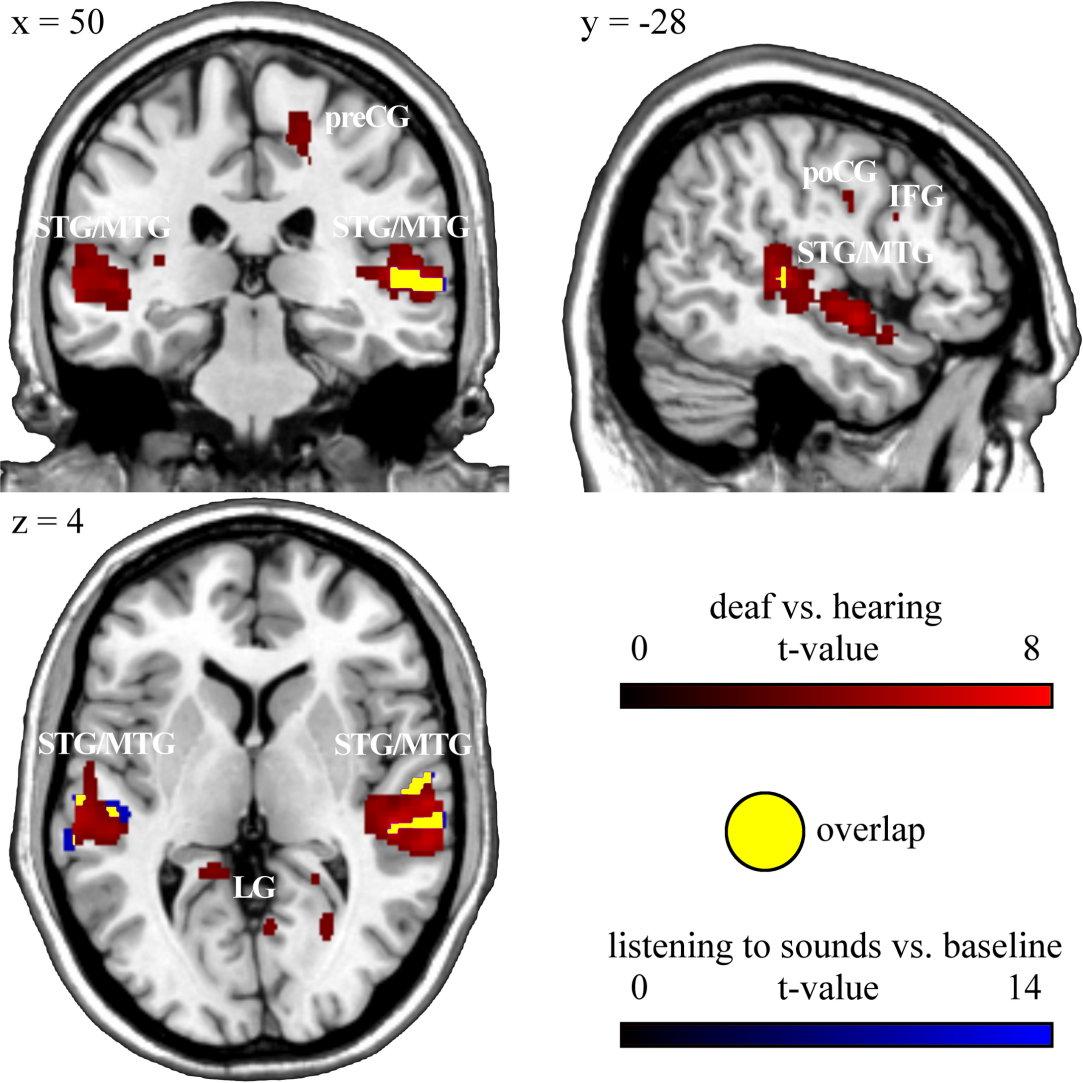
Functional-anatomical overlap between activation during the animacy decision task in deaf versus hearing participants (p < 0.001, uncorrected) and activity to sounds versus baseline in hearing participants (p < 0.05, FWE corrected). A juxtaposition of the activation pattern in deaf vs. hearing participants in the conceptual task with the activation pattern for sound listening vs. baseline in hearing participants reveals an overlap in superior and middle temporal gyrus. This indicates neuroplastic changes in auditory brain areas of deaf participants. STG: superior temporal gyrus, MTG: middle temporal gyrus, preCG: precentral gyrus, poCG: postcentral gyrus, IFG: inferior frontal gyrus, LG: lingual gyrus.

Listening to acoustic stimuli compared to baseline significantly increased the MR signal in hearing participant’s bilateral superior and left middle temporal gyrus corresponding to BA 21, 22 and 48 (Fig 4, Table 3).

### Functional overlap across tasks

As superior and middle temporal areas, the auditory cortex in hearing people, might be functionally reorganized in deaf individuals, we performed masking analyses to determine functional overlap of activity within this region across the different tasks employed in our study. Inclusive masking analyses (Table 4) showed that the same temporal brain region activated during animacy decision in deaf versus hearing participants (superior/middle temporal gyrus, BA 21/22) was also activated when they viewed GSL signs compared to baseline (Fig 2). This temporal region identified in deaf participants was also active in hearing participants when listening to sounds compared to baseline (Fig 4). In order to determine whether in deaf participants this temporal brain region was specifically activated for GSL signs, we inclusively masked activity to sign language versus baseline in deaf participants with activity during (non-verbal) action observation versus baseline in deaf participants (Fig 3 and Table 4). Masking analysis revealed overlapping brain activation mainly within occipital brain regions (BA 19), inferior frontal gyrus pars opercularis (BA 44) and inferior/middle temporal gyrus (BA 37), but not within superior and middle temporal gyrus (BA 21, 22). The specificity of superior and middle temporal brain activation in deaf people was additionally confirmed by exclusively masking activity to sign language versus baseline in deaf participants with activity during action observation versus baseline in deaf (S2 Table). Exclusive masking showed that superior and middle temporal gyrus is specifically active in deaf participants during viewing GSL signs, but not during non-verbal action observation. Exclusive masking activity of deaf versus hearing participants in the animacy decision task with activity to sign language compared to baseline in deaf subjects revealed clusters in left middle temporal and right superior temporal gyrus as well as in occipital (bilateral lingual gyrus, bilateral calcarine sulcus and left cuneus, BA 18/19) and in frontal areas (right precentral gyrus, BA 4/6 and right pars opercularis, BA 44) to be specifically activated in the animacy decision task (S2 Table). This shows that, within deaf people`s temporal cortex, more medial aspects within the temporal plane were only activated during animacy decision, whereas overlapping activity with sign language observation was confined to lateral temporal cortex. Furthermore, activation in occipital areas and in frontal areas was specific for the conceptual animacy decision task. However, in particular these activation differences within temporal cortex are difficult to interpret due to the different task demands in the animacy decision task and sign language observation.

**Table 4:** Inclusive masking analyses.

Brain region	BA	MNI coordinates (mm)	T	P_Voxel_ _FWE-corr_	Cluster size	P_Cluster_ _FWE-corr_
***Contrast deaf vs*. *hearing in the animacy decision task inclusively masked with activity to sounds vs*. *baseline in hearing subjects***						
Superior temporal R	22	50–28 4	6.16	< 0.0001	62	0.001
Superior temporal R	21	62–28 2	5.49	< 0.0001		
Superior temporal L	22	-50–6–8	5.93	< 0.0001	37	0.004
Superior temporal L	48	-56–10–4	3.97	0.015		
Middle temporal L	21	-58–22–4	5.59	< 0.0001	87	< 0.0001
Middle temporal L	21	-48–26 0	4.66	< 0.0001		
***Contrast deaf vs*. *hearing in the animacy decision task inclusively masked with activity to sign language vs*. *baseline in deaf subjects***						
Middle temporal R	21	60–20–6	6.44	0.001	539	< 0.0001
Middle temporal R	21	62–34 4	6.14	0.002		
Superior temporal R	21	52 0–12	5.97	0.003		
Middle temporal L	21	-62–24 2	6.04	0.003	428	< 0.0001
Superior temporal L	21	-50–6–10	5.50	0.011		
Superior temporal L	22	-58–32 12	5.42	0.013		
***Activity to sign language vs*. *baseline in deaf subjects inclusively masked with activity to action observation vs*. *baseline in deaf subjects***						
Inferior temporal R	37	52–72–2	17.96	< 0.0001	562	< 0.0001
Inferior occipital R	19	46–74–12	12.86	< 0.0001		
Middle temporal L	37	-56–62 2	14.76	< 0.0001	536	< 0.0001
Middle occipital L	19	-48–80 0	13.53	< 0.0001		
Inferior occipital L	19	-46–74–12	12.24	< 0.0001		
Inferior frontal pars opercularis L	44	-50 12 24	13.33	< 0.0001	30	0.008
Superior temporal L	42	-62–38 18	10.17	< 0.0001	85	0.001

P-values at voxel and cluster level are FWE corrected. Shown are peak voxels with highest t-values for significant clusters and their local maxima more than 8 mm apart. BA: Brodmann Area, MNI: Montréal Neurological Institute, R: right, L: left. See also S2 Table.

## Discussion

The present study indicated neuro-plastic changes in the conceptual system of early onset deaf individuals: Compared with hearing participants, conceptual processing during the animacy decision task in deaf individuals more strongly activated superior and middle temporal gyri. In hearing people, this region compasses auditory cortex [58] as also shown by the anatomical overlap with the activation pattern of the sound listening experiment in this participant group. In deaf individuals, temporal cortex was not only involved during animacy decision, but was also recruited by viewing lexical GSL signs. In line with earlier studies [1,21,22,23,24], our findings indicate a functional reorganization of auditory cortex in deaf people, which becomes sensitive to sign language instead of spoken language.

In line with earlier work [24], our results also show that these neural circuits in temporal cortex of deaf individuals activated by animacy decision and sign language were not generally responsive to visual or visuo-motor processing, because viewing non-verbal hand actions mainly elicited activity outside temporal cortex in occipto-parietal and frontal areas, quite comparable to hearing individuals. Observing hand actions activated in deaf participants only a small cluster in right superior temporal cortex (BA 42), indicative of some general cross-modal plasticity [12,24]. However, this cluster failed to reach significance in the direct comparison with hearing participants. Although the actor in the video of the action observation experiment was different from the actor in the video of the sign language observation experiment, it is difficult to explain why such comparably subtle changes in the visual stimulation would produce these pronounced differences in brain activation. In comparison, the entirely different stimulation in the animacy decision task (static pictures of common objects) yielded an overlapping activation with sign language observation in temporal cortex. For that reason, it is appropriate to assume that the type of processing (verbal vs. non-verbal) is the relevant factor for obtaining superior temporal activation in deaf participants and not perceptual aspects of the visual stimuli themselves.

If greater temporal cortex activation in the deaf than in the hearing group reflected distraction of hearing individuals originating from the perception of scanner noise, which was absent in deaf participants, comparable group differences in brain activity should also be found for action observation. However, brain activity during action observation was comparable for deaf and hearing participants. Furthermore, when considering the activity pattern within the deaf group, activity in left superior and middle temporal cortex, a prominent activation focus during the conceptual task and sign language observation, was entirely absent in action observation. These two observations rule out the possibility that group differences were simply driven by the perception of scanner noise in hearing participants and its absence in deaf participants.

We therefore assume that the considerable overlap of activity within superior and middle temporal cortex during animacy decision and sign language observation in deaf individuals indexes a contribution of the language system to conceptual processing. Thus, stronger activations in deaf compared to hearing individuals in superior and middle temporal gyri most likely reflect stronger activation of language representations associated with the depicted object, in order to facilitate animacy decision. As outlined in more detail below, the lack of auditory information as basis for conceptual knowledge might not be appropriately compensated through visual or motor channels. Object categorization might thus be supported by elaborated access to verbal associations stored within language areas [39].

It should be mentioned at this point, that, despite that our deaf participants all reported that GSL is their native language, they did not have signing parents. Thus, it is possible that their early access to GSL was restricted and that they were delayed in their language development. Neuro-plastic changes in temporal cortex of deaf participants may thus not only be influenced by auditory deprivation, but also by language delay (for a discussion see [59]).

Finally, it is noteworthy that superior temporal cortex activation has not only been observed in deaf individuals during conceptual and language tasks. Posterior superior temporal cortex activation has been found also during verbal and non-verbal visual working memory tasks [14]. It is therefore possible that this region plays a general role in various forms of higher-level cognition in deaf people due to its functional reorganization. Alternatively, superior temporal cortex activation might reflect language processing relevant for working memory maintenance of verbal and non-verbal material [14] and for conceptual processing during animacy decisions.

Additionally to superior and middle temporal areas, animacy decisions in deaf compared to hearing participants elicited a higher MR signal in visual regions in occipital cortex as well as in motor regions in frontal and parietal cortex. These neural activations in visual and motor cortex may relate to cognitive activation of sign language representations [60]. However, regarding earlier work on the grounded cognition theory [46,61], these regions in visual and motor cortex presumably code visual and action-related conceptual knowledge, respectively.

The stronger recruitment of visual and motor areas at the neural level was in line with the more frequent report of visual and motor features in deaf than in hearing participants at the behavioral level, when reporting object properties. Deaf participants reported properties in written language, i.e. not in their first language GSL. Although the use of foreign language may reduce mental imagery [62], it is unlikely that differences in nativeness or language proficiency have influenced the result pattern in the present property listing task. The property listing task assesses the semantic content of concepts, but does not necessarily involve imagery [6]. Furthermore, deaf participants produced even more (not less) visual and motor features than hearing participants indicating that access to sensory-motor knowledge was not generally incurred in this participant group due to written language task.

Our results suggest an experience-dependent compensatory change in the grounding of concepts in the sensory and motor systems: The loss of the auditory channel may be partially compensated by an increased importance of visual and motor channels for acquiring object knowledge, in agreement with the documented enhanced sensory performance of deaf people in the intact modalities [3]. As a consequence, conceptual representations in deaf people may be more strongly grounded in the visual and motor brain systems compared with hearing people. In addition, the use of sign language in verbal communication might have emphasized the importance of visuo-motor features of concepts in deaf participants because signforms may readily evoke mental images [63] or may increase the salience of animacy properties [64]. However, we can rule out the possibility that greater visual and motor activation in deaf individuals during the conceptual animacy decision task simply reflects unspecific cortical reorganization, because comparable group differences were not observed for non-verbal action observation (see also the discussion above).

Interestingly, deaf participants also reported auditory object properties in the property listing task, albeit less frequently than hearing participants. Obviously, the meaning of these sound-related words cannot be based on the personal sensory experience of deaf people. The use of words, whose meaning lack personal sensory experience, is known as ‘verbalism’, and has been originally described in the blind [42]. This phenomenon indexes in the present context that conceptual processing in deaf people also relies on sound knowledge rooted in verbal associations. Deaf people may acquire sound-related words associated with objects from movies or from written texts, in which sound experiences of hearing people are verbally described [65]. This indicates that conceptual processing in deaf people might be more strongly supported by the language system compared with hearing people: Although deaf participants showed an enhanced recruitment of visual and motor regions compared with hearing participants, the lacking auditory conceptual memory traces might not be appropriately compensated through visual or motor channels alone and might require elaborated access to verbal associations stored within language areas [39]. This may include activation of sound words associated with the objects, in agreement with the above mentioned phenomenon of sound word verbalism [42].

The presently observed experience-dependent neuroplastic changes of the conceptual brain systems in deaf individuals reveal a conjoint contribution of the language and sensory-motor brain systems to conceptual processing. Our results strongly support variants of grounded cognition theories, which propose that activation of verbal associations in language areas are complemented by modality-specific processing in sensory-motor areas [11,39,40].

In contrast to our findings in deaf individuals, earlier studies in blind people did not observe functional plasticity of the conceptual system [52,53]. It has therefore been argued that the functional neuroarchitecture of the conceptual system does not require sensory experience. We assume that this discrepancy reflects the fact that deafness results in a more dramatic change of the interactions with the environment compared with blindness for two reasons: In deafness, the brain has to adopt to sign language, with quite different physical and linguistic properties compared with spoken language [22]. This also constrains social interactions with hearing people. Furthermore, the missing sound experience for gaining object knowledge cannot be as adequately compensated by other sensory channels as in blindness, because touch can replace vision for many object attributes such as form or texture [53].

In conclusion, the present study provides important evidence for experience-dependent plasticity of the conceptual system at the behavioral and neural level. We showed that early onset deafness induces plastic alterations of the functional neuroanatomy of the conceptual system: Compared with hearing people, the conceptual system of deaf individuals is more deeply rooted in the language system, complemented by enhanced grounding in visual and motor brain regions. This emphasizes the importance of sign language for higher-level cognition in deaf people. As concepts are the main building blocks of human thought, the present findings give insight how experience shapes conceptual brain circuits and thus the way individuals conceive the world.

## Supporting information

S1 Table Activation peaks for main contrasts in the animacy decision task (activation during animacy decision in deaf/hearing subjects vs. baseline). The statistical threshold was set to p < 0.05 FWE corrected for the whole brain (cluster size ≥ 30 voxel). Shown are peak voxels with highest t-values for significant clusters and their local maxima more than 8 mm apart. For clusters comprising more than 10000 voxels embedded local maxima more than 4 mm apart are listed (grey font) in order to provide a more differentiated characterization of the activated brain regions. BA: Brodmann Area, MNI: Montréal Neurological Institute, R: right, L: left.
(DOCX)

S2 Table Exclusive masking analyses. Shown are peak voxels with highest t-values for significant clusters and their local maxima more than 8 mm apart. * p-value also significant for FWE correction, BA: Brodmann Area, MNI: Montréal Neurological Institute, R: right, L: left.
(DOCX)

## References

[1] Campbell R, MacSweeney MA, Waters D (2008) Sign language and the brain: A review. Journal of Deaf Studies and Deaf Education 13: 3–20. doi: 10.1093/deafed/enm035 17602162 10.1093/deafed/enm035

[2] Bavelier D, Hirshorn EA (2010) I see where you're hearing: how cross-modal plasticity may exploit homologous brain structures. Nature Neuroscience 13: 1309–1311. doi: 10.1038/nn1110-1309 20975752 10.1038/nn1110-1309

[3] Neville HJ (1990) Intermodal competition and compensation in development—Evidence from studies of the visual system in congenitally deaf adults. Annals of the New York Academy of Sciences 608: 71–91. 2075968 10.1111/j.1749-6632.1990.tb48892.x

[4] Levelt WJ, Roelofs A, Meyer AS (1999) A theory of lexical access in speech production. Behavioral and Brain Sciences 22: 1–75. 11301520 10.1017/s0140525x99001776

[5] Humphreys GW, Riddoch MJ, Quinlan PT (1988) Cascade processes in picture identification. Cognitive Neuropsychology 5: 67–103.

[6] Kiefer M, Barsalou LW (2013) Grounding the human conceptual system in perception, action, and internal states In: Prinz W, Beisert M, Herwig A, editors. Action science: Foundations of an emerging discipline. Cambridge: MIT Press pp. 381–407.

[7] Kiefer M (2001) Perceptual and semantic sources of category-specific effects: Event-related potentials during picture and word categorization. Memory & Cognition 29: 100–116. 11277454 10.3758/bf03195745

[8] McRae K, de Sa VR, Seidenberg MS (1997) On the nature and scope of featural representations of word meaning. Journal of Experimental Psychology: General 126: 99–130. 9163932 10.1037//0096-3445.126.2.99

[9] Humphreys GW, Price CJ, Riddoch MJ (1999) From objects to names: A cognitive neuroscience approach. Psychological Research 62: 118–130. 10472198 10.1007/s004260050046

[10] Pulvermüller F (1999) Words in the brain's language. Behavioral and Brain Sciences 22: 253–336. 11301524

[11] Kiefer M, Pulvermüller F (2012) Conceptual representations in mind and brain: Theoretical developments, current evidence and future directions. Cortex 48: 805–825. doi: 10.1016/j.cortex.2011.04.006 21621764 10.1016/j.cortex.2011.04.006

[12] Vachon P, Voss P, Lassonde M, Leroux JM, Mensour B, et al (2013) Reorganization of the auditory, visual and multimodal areas in early deaf individuals. Neuroscience 245: 50–60. doi: 10.1016/j.neuroscience.2013.04.004 23590908 10.1016/j.neuroscience.2013.04.004

[13] Bavelier D, Brozinsky C, Tomann A, Mitchell T, Neville H, et al (2001) Impact of early deafness and early exposure to sign language on the cerebral organization for motion processing. The Journal of Neuroscience 21: 8931–8942. 11698604 10.1523/JNEUROSCI.21-22-08931.2001 PMC6762265

[14] Cardin V, Rudner M, De Oliveira RF, Andin J, M TS, et al (2017) The organization of working memory networks is shaped by early sensory experience. Cerebral Cortex: 1–15. 10.1093/cercor/bhx222 28968707

[15] Rudner M, Karlsson T, Gunnarsson J, Rönnberg J (2013) Levels of processing and language modality specificity in working memory. Neuropsychologia 51: 656–666. doi: 10.1016/j.neuropsychologia.2012.12.011 23287569 10.1016/j.neuropsychologia.2012.12.011

[16] Cardin V, Orfanidou E, Kästner L, Rönnberg J, Woll B, et al (2016) Monitoring Different Phonological Parameters of Sign Language Engages the Same Cortical Language Network but Distinctive Perceptual Ones. Journal of Cognitive Neuroscience 28: 20–40. doi: 10.1162/jocn_a_00872 26351993 10.1162/jocn_a_00872

[17] MacSweeney M, Waters D, Brammer MJ, Woll B, Goswami U (2008) Phonological processing in deaf signers and the impact of age of first language acquisition. NeuroImage 40: 1369–1379. doi: 10.1016/j.neuroimage.2007.12.047 18282770 10.1016/j.neuroimage.2007.12.047 PMC2278232

[18] Röder B, Teder-Salejarvi W, Sterr A, Rosler F, Hillyard SA, et al (1999) Improved auditory spatial tuning in blind humans. Nature 400: 162–166. doi: 10.1038/22106 10408442 10.1038/22106

[19] Voss P, Gougoux F, Zatorre RJ, Lassonde M, Lepore F (2008) Differential occipital responses in early- and late-blind individuals during a sound-source discrimination task. NeuroImage 40: 746–758. doi: 10.1016/j.neuroimage.2007.12.020 18234523 10.1016/j.neuroimage.2007.12.020

[20] Rauschecker JP, Korte M (1993) Auditory compensation for early blindness in cat cerebral cortex. The Journal of Neuroscience 13: 4538–4548. 8410202 10.1523/JNEUROSCI.13-10-04538.1993 PMC6576362

[21] Petitto LA, Zatorre RJ, Gauna K, Nikelski EJ, Dostie D, et al (2000) Speech-like cerebral activity in profoundly deaf people processing signed languages: Implications for the neural basis of human language. Proceedings of the National Academy of Sciences of the United States of America 97: 13961–13966. doi: 10.1073/pnas.97.25.13961 11106400 10.1073/pnas.97.25.13961 PMC17683

[22] MacSweeney M, Woll B, Campbell R, McGuire PK, David AS, et al (2002) Neural systems underlying British Sign Language and audio-visual English processing in native users. Brain 125: 1583–1593. 12077007 10.1093/brain/awf153

[23] Neville HJ, Bavelier D, Corina D, Rauschecker J, Karni A, et al (1998) Cerebral organization for language in deaf and hearing subjects: Biological constraints and effects of experience. Proceedings of the National Academy of Sciences of the United States of America 95: 922–929. 9448260 10.1073/pnas.95.3.922 PMC33817

[24] Cardin V, Orfanidou E, Ronnberg J, Capek CM, Rudner M, et al (2013) Dissociating cognitive and sensory neural plasticity in human superior temporal cortex. Nature Communications 4: 1473 doi: 10.1038/ncomms2463 23403574 10.1038/ncomms2463

[25] Anderson JR (1983) The architecture of cognition. Hillsdayle, NJ: Lawrence Erlbaum Associates, Inc.

[26] Caramazza A, Mahon BZ (2003) The organization of conceptual knowledge: The evidence from category-specific semantic deficits. Trends in Cognitive Sciences 7: 354–361. 12907231 10.1016/s1364-6613(03)00159-1

[27] McClelland JL, Rogers TT (2003) The parallel distributed processing approach to semantic cognition. Nature Reviews Neuroscience 4: 310–322. doi: 10.1038/nrn1076 12671647 10.1038/nrn1076

[28] Tyler LK, Moss HE (2001) Towards a distributed account of conceptual knowledge. Trends in Cognitive Sciences 5: 244–252. 11390295 10.1016/s1364-6613(00)01651-x

[29] Pobric G, Jefferies E, Lambon Ralph MA (2010) Amodal semantic representations depend on both anterior temporal lobes: Evidence from repetitive transcranial magnetic stimulation. Neuropsychologia 48: 1336–1342. doi: 10.1016/j.neuropsychologia.2009.12.036 20038436 10.1016/j.neuropsychologia.2009.12.036

[30] de Zubicaray GI, Wilson SJ, McMahon KL, Muthiah S (2001) The semantic interference effect in the picture-word paradigm: An event-related fMRI study employing overt responses. Human Brain Mapping 14: 218–227. 11668653 10.1002/hbm.1054 PMC6871995

[31] Hoffman P, Pobric G, Drakesmith M, Lambon Ralph MA (2011) Posterior middle temporal gyrus is involved in verbal and non-verbal semantic cognition: Evidence from rTMS. Aphasiology 26: 1119–1130.

[32] Pulvermüller F, Fadiga L (2010) Active perception: Sensorimotor circuits as a cortical basis for language. Nature Reviews Neuroscience 11: 351–360. doi: 10.1038/nrn2811 20383203 10.1038/nrn2811

[33] Gallese V, Lakoff G (2005) The brain's concepts: The role of the sensory-motor system in conceptual knowledge. Cognitive Neuropsychology 22: 455–479. doi: 10.1080/02643290442000310 21038261 10.1080/02643290442000310

[34] Barsalou LW (2008) Grounded cognition. Annual Review of Psychology 59: 617–645. doi: 10.1146/annurev.psych.59.103006.093639 17705682 10.1146/annurev.psych.59.103006.093639

[35] Martin A (2007) The representation of object concepts in the brain. Annual Review of Psychology 58: 25–45. doi: 10.1146/annurev.psych.57.102904.190143 16968210 10.1146/annurev.psych.57.102904.190143

[36] Hoenig K, Müller C, Herrnberger B, Spitzer M, Ehret G, et al (2011) Neuroplasticity of semantic maps for musical instruments in professional musicians. NeuroImage 56: 1714–1725. doi: 10.1016/j.neuroimage.2011.02.065 21356317 10.1016/j.neuroimage.2011.02.065

[37] Kiefer M, Sim E-J, Liebich S, Hauk O, Tanaka JW (2007) Experience-dependent plasticity of conceptual representations in human sensory-motor areas. Journal of Cognitive Neuroscience 19: 525–542. doi: 10.1162/jocn.2007.19.3.525 17335399 10.1162/jocn.2007.19.3.525

[38] Beilock SL, Lyons IM, Mattarella-Micke A, Nusbaum HC, Small SL (2008) Sports experience changes the neural processing of action language. Proceedings of the National Academy of Sciences, USA 105: 13269–13273. 10.1073/pnas.0803424105 PMC2527992 18765806

[39] Barsalou LW, Santos A, Simmons WK, Wilson CD (2008) Language and simulation in conceptual processing. In: De Vega M, Glenberg AM, Graesser AC, editors. Symbols, embodiment, and meaning Oxford: Oxford University Press.

[40] Simmons WK, Hamann SB, Harenski CL, Hu XP, Barsalou LW (2008) fMRI evidence for word association and situated simulation in conceptual processing. Journal of Physiology (Paris) 102: 106–119. doi: 10.1016/j.jphysparis.2008.03.014 18468869 10.1016/j.jphysparis.2008.03.014

[41] Rogers TT, Lambon Ralph MA, Garrard P, Bozeat S, McClelland JL, et al (2004) Structure and deterioration of semantic memory: A neuropsychological and computational investigation. Psychological Review 111: 205–235. doi: 10.1037/0033-295X.111.1.205 14756594 10.1037/0033-295X.111.1.205

[42] Chauvey V, Hatwell Y, Verine B, Kaminski G, Gentaz E (2012) Lexical references to sensory modalities in verbal descriptions of people and objects by congenitally blind, late blind and sighted adults. Plos One 7. 10.1371/journal.pone.0044020 PMC3431324 22956997

[43] Trumpp NM, Kliese D, Hoenig K, Haarmaier T, Kiefer M (2013) Losing the sound of concepts: Damage to auditory association cortex impairs the processing of sound-related concepts. Cortex 49: 474–486. doi: 10.1016/j.cortex.2012.02.002 22405961 10.1016/j.cortex.2012.02.002

[44] Kiefer M, Sim E-J, Herrnberger B, Grothe J, Hoenig K (2008) The sound of concepts: Four markers for a link between auditory and conceptual brain systems. The Journal of Neuroscience 28: 12224–12230. doi: 10.1523/JNEUROSCI.3579-08.2008 19020016 10.1523/JNEUROSCI.3579-08.2008 PMC6671691

[45] Kiefer M (2005) Repetition priming modulates category-related effects on event-related potentials: Further evidence for multiple cortical semantic systems. Journal of Cognitive Neuroscience 17: 199–211. doi: 10.1162/0898929053124938 15811233 10.1162/0898929053124938

[46] Hoenig K, Sim E-J, Bochev V, Herrnberger B, Kiefer M (2008) Conceptual flexibility in the human brain: Dynamic recruitment of semantic maps from visual, motion and motor-related areas. Journal of Cognitive Neuroscience 20: 1799–1814. doi: 10.1162/jocn.2008.20123 18370598 10.1162/jocn.2008.20123

[47] Warrington EK, McCarthy R (1987) Categories of knowledge. Brain 110: 1273–1296. 3676701 10.1093/brain/110.5.1273

[48] Martin A, Wiggs CL, Ungerleider LG, Haxby JV (1996) Neural correlates of category-specific knowledge. Nature 379: 649–652. doi: 10.1038/379649a0 8628399 10.1038/379649a0

[49] James TW, Gauthier I (2003) Auditory and action semantic features activate sensory-specific perceptual brain regions. Current Biology 13: 1792–1796. 14561404 10.1016/j.cub.2003.09.039

[50] Weisberg J, van Turennout M, Martin A (2007) A neural system for learning about object function. Cerebral Cortex 17: 513–521. doi: 10.1093/cercor/bhj176 16581980 10.1093/cercor/bhj176 PMC1817810

[51] Bellebaum C, Tettamanti M, Marchetta E, Della Rosa P, Rizzo G, et al (2013) Neural representations of unfamiliar objects are modulated by sensorimotor experience. Cortex 49: 1110–1125. doi: 10.1016/j.cortex.2012.03.023 22608404 10.1016/j.cortex.2012.03.023

[52] Noppeney U, Friston KJ, Price CJ (2003) Effects of visual deprivation on the organization of the semantic system. Brain 126: 1620–1627. doi: 10.1093/brain/awg152 12805112 10.1093/brain/awg152

[53] Mahon BZ, Anzellotti S, Schwarzbach J, Zampini M, Caramazza A (2009) Category-specific organization in the human brain does not require visual experience. Neuron 63: 397–405. doi: 10.1016/j.neuron.2009.07.012 19679078 10.1016/j.neuron.2009.07.012 PMC2743253

[54] Oldfield R (1971) The assessment and analysis of handedness: The Edinburgh Inventory. Neuropsychologia 9: 97–113. 5146491 10.1016/0028-3932(71)90067-4

[55] Horn W (1983) Leistungsprüfsystem: L-P-S. Göttingen [u.a.]: Verl. für Psychologie, Hogrefe.

[56] Sim EJ, Helbig HB, Graf M, Kiefer M (2015) When action observation facilitates visual perception: Activation in visuo-motor areas contributes to object recognition. Cerebral Cortex 25: 2907–2918. doi: 10.1093/cercor/bhu087 24794918 10.1093/cercor/bhu087

[57] Ashburner J (2007) A fast diffeomorphic image registration algorithm. NeuroImage 38: 95–113. doi: 10.1016/j.neuroimage.2007.07.007 17761438 10.1016/j.neuroimage.2007.07.007

[58] Nieuwenhuys R, Voogd J, Huijzen van C (2008) The human central nervous system. Berlin: Springer.

[59] MacSweeney M, Cardin V (2015) What is the function of auditory cortex without auditory input? Brain 138: 2468–2470. doi: 10.1093/brain/awv197 26304150 10.1093/brain/awv197 PMC4920248

[60] Emmorey K, McCullough S, Mehta S, Grabowski TJ (2014) How sensory-motor systems impact the neural organization for language: direct contrasts between spoken and signed language. Frontiers in Psychology 5. 10.3389/fpsyg.2014.00484 PMC4033845 24904497

[61] Chao LL, Haxby JV, Martin A (1999) Attribute-based neural substrates in temporal cortex for perceiving and knowing about objects. Nature Neuroscience 2: 913–919. doi: 10.1038/13217 10491613 10.1038/13217

[62] Hayakawa S, Keysar B (2018) Using a foreign language reduces mental imagery. Cognition 173: 8–15. doi: 10.1016/j.cognition.2017.12.010 29278805 10.1016/j.cognition.2017.12.010

[63] Vigliocco G, Vinson DP, Woolfe T, Dye MWG, Woll B (2005) Language and imagery: effects of language modality. Proceedings of the Royal Society B: Biological Sciences 272: 1859–1863. doi: 10.1098/rspb.2005.3169 16096100 10.1098/rspb.2005.3169 PMC1559869

[64] Meir I, Aronoff M, Börstell C, Hwang S-O, Ilkbasaran D, et al (2017) The effect of being human and the basis of grammatical word order: Insights from novel communication systems and young sign languages. Cognition 158: 189–207. doi: 10.1016/j.cognition.2016.10.011 27837693 10.1016/j.cognition.2016.10.011

[65] Bream PB (1992) Einführung in die Gebärdensprache und ihre Erforschung; S. P, editor. Hamburg: Signum-Verlag. 232 p.

