# Supplementary material for: Functional reorganization of the conceptual brain system after deafness in early childhood
Source: PLoS One. 2018 Jul 5;13(7):e0198894. doi: 10.1371/journal.pone.0198894 (PMC6033386; doi:10.1371/journal.pone.0198894)
Supplement: S1 Table — The statistical threshold was set to p < 0.05 FWE corrected for the whole brain (cluster size ≥ 30 voxel). Shown are peak voxels with highest t-values for significant clusters and their local maxima more than 8 mm apart. For clusters comprising more than 10000 voxels embedded local maxima more than 4 mm apart are listed (grey font) in order to provide a more differentiated characterization of the activated brain regions. BA: Brodmann Area, MNI: Montréal Neurological Institute, R: right, L: left. (DOCX) [file pone.0198894.s001.docx]

**Supplementary Information**

Functional reorganization of the conceptual brain system after deafness in early childhood

Natalie M. Trumpp and Markus Kiefer

Ulm University, Department of Psychiatry, Ulm, Germany

**S1 Table.** **Activation peaks for main contrasts in the animacy decision task** (activation during animacy decision in deaf/hearing subjects vs. baseline).

| **Brain region** | **BA** | **MNI coordinates (mm)** | **T** | **P_Voxel_** | **Cluster size** | **P_Cluster_** |
| --- | --- | --- | --- | --- | --- | --- |
| ***deaf subjects*** | | | | | | |
| Middle temporal L | 48 | -32 -20 0 | 14.31 | < 0.0001 | 57153 | < 0.0001 |
| *Vermis* |  | *4 -56 -14* | *14.14* | *< 0.0001* |  |  |
| *Thalamus L* |  | *-12 -24 -2* | *13.91* | *< 0.0001* |  |  |
| *Fusiform L* | *37* | *-36 -58 -8* | *13.74* | *< 0.0001* |  |  |
| *Putamen L* |  | *-30 -14 10* | *13.51* | *< 0.0001* |  |  |
| *Cerebellum R* |  | *10 -52 -14* | *13.38* | *< 0.0001* |  |  |
| *Inferior parietal R* | *40* | *36 -42 44* | *13.32* | *< 0.0001* |  |  |
| *Hippocampus L* | *20* | *-40 -18 -10* | *13.27* | *< 0.0001* |  |  |
| *Cerebellum L* |  | *-12 -60 -16* | *12.95* | *< 0.0001* |  |  |
| *Fusiform L* | *19* | *-40 -64 -16* | *12.94* | *< 0.0001* |  |  |
| *Calcarine R* | *18* | *4 -70 18* | *12.83* | *< 0.0001* |  |  |
| *Inferior occipital R* | *37* | *42 -62 -14* | *12.71* | *< 0.0001* |  |  |
| *Pallidum R* |  | *16 0 8* | *12.7* | *< 0.0001* |  |  |
| *Superior temporal pole R* | *38* | *56 12 0* | *12.39* | *< 0.0001* |  |  |
| *Superior temporal L* | *42* | *-56 -30 14* | *12.35* | *< 0.0001* |  |  |
| *Inferior frontal pars opercularis R* | *44* | *58 12 22* | *12.09* | *< 0.0001* |  |  |
| *Inferior occipital L* | *19* | *-42 -70 -10* | *12.03* | *< 0.0001* |  |  |
| *Superior temporal R* | *22* | *54 -34 8* | *11.97* | *< 0.0001* |  |  |
| *Precentral R* | *44* | *50 12 34* | *11.76* | *< 0.0001* |  |  |
| *Thalamus R* |  | *12 -10 10* | *11.76* | *< 0.0001* |  |  |
| *Insula R* | *48* | *36 -2 8* | *11.75* | *< 0.0001* |  |  |
| Inferior frontal pars triangularis L | 45 | -34 34 14 | 11.14 | < 0.0001 | 1205 | < 0.0001 |
| Middle frontal L | 10 | -36 50 12 | 9.43 | < 0.0001 |  |  |
| Middle orbito-frontal R | 47 | 32 48 -4 | 7.44 | 0.001 | 33 | < 0.0001 |
| Anterior cingulum R | 24 | 6 38 10 | 6.78 | 0.004 | 10 | 0.002 |
| ***hearing subjects*** | | | | | | |
| Middle temporal L | 48 | -30 -20 2 | 12.80 | < 0.0001 | 20442 | < 0.0001 |
| *Thalamus R* |  | *18 -10 14* | *11.28* | *< 0.0001* |  |  |
| *Vermis* |  | *4 -54 -14* | *11.18* | *< 0.0001* |  |  |
| *Fusiform L* | *37* | *-36 -56 -14* | *11.06* | *< 0.0001* |  |  |
| *Insula L* | *48* | *-36 16 -6* | *10.94* | *< 0.0001* |  |  |
| *Pallidum L* |  | *-18 8 2* | *10.93* | *< 0.0001* |  |  |
| *Hippocampus L* | *27* | *-18 -30 -2* | *10.87* | *< 0.0001* |  |  |
| *Putamen L* |  | *-34 -10 -4* | *10.83* | *< 0.0001* |  |  |
| *Pallidum R* |  | *16 0 8* | *10.49* | *< 0.0001* |  |  |
| *Fusiform R* | *37* | *26 -42 -18* | *10.46* | *< 0.0001* |  |  |
| *Caudate L* |  | *-12 6 10* | *10.45* | *< 0.0001* |  |  |
| *Caudate R* |  | *12 8 6* | *10.43* | *< 0.0001* |  |  |
| *Thalamus L* |  | *-14 -20 -4* | *10.40* | *< 0.0001* |  |  |
| *Inferior temporal R* | *19* | *48 -70 -4* | *10.30* | *< 0.0001* |  |  |
| *Insula R* | *48* | *42 -60 -14* | *10.29* | *< 0.0001* |  |  |
| *Hippocampus R* | *27* | *22 -28 -6* | *10.14* | *< 0.0001* |  |  |
| *Middle occipital L* | *37* | *-46 -68 0* | *10.10* | *< 0.0001* |  |  |
| *Putamen R* | *48* | *34 -10 -8* | *10.05* | *< 0.0001* |  |  |
| Supramarginal R | 2 | 44 -36 46 | 11.19 | < 0.0001 | 2657 | < 0.0001 |
| Inferior parietal R | 40 | 36 -46 42 | 9.39 | < 0.0001 |  |  |
| Angular R | 7 | 28 -60 40 | 9.37 | < 0.0001 |  |  |
| Middle cingulum L |  | -2 22 36 | 10.98 | < 0.0001 | 3484 | < 0.0001 |
| Anterior cingulum L |  | -4 10 28 | 9.46 | < 0.0001 |  |  |
| Supplementory motor area L | 6 | -2 -4 64 | 9.09 | < 0.0001 |  |  |
| Rolandic operculum L | 48 | -46 -24 16 | 10.81 | < 0.0001 | 4046 | < 0.0001 |
| Inferior parietal L | 2 | -44 -32 40 | 10.57 | < 0.0001 |  |  |
| Postcentral L | 2 | -50 -32 50 | 9.59 | < 0.0001 |  |  |
| Precentral L | 6 | -26 -14 60 | 8.97 | < 0.0001 | 330 | < 0.0001 |
| Precuneus R | 5 | 6 -42 54 | 7.71 | < 0.0001 | 71 | < 0.0001 |
| Middle cingulum R |  | 16 -34 50 | 6.90 | 0.003 |  |  |
| Middle frontal L | 46 | -32 42 26 | 7.49 | 0.001 | 158 | < 0.0001 |
| Inferior frontal pars triangularis L | 45 | -32 36 12 | 6.52 | 0.007 |  |  |
| Superior temporal L | 42 | -58 -42 22 | 6.63 | 0.006 | 21 | < 0.0001 |

The statistical threshold was set to p < 0.05 FWE corrected for the whole brain (cluster size ≥ 10 voxel). For clusters comprising more than 10000 voxels embedded local maxima more than 4 mm apart are listed (grey font) in order to provide a more differentiated characterization of the activated brain regions. Shown are cluster and peak voxels with highest t-values. BA: Brodmann Area, MNI: Montréal Neurological Institute, R: right, L: left.
