# Supplementary material for: Functional reorganization of the conceptual brain system after deafness in early childhood
Source: PLoS One. 2018 Jul 5;13(7):e0198894. doi: 10.1371/journal.pone.0198894 (PMC6033386; doi:10.1371/journal.pone.0198894)
Supplement: S2 Table — Shown are peak voxels with highest t-values for significant clusters and their local maxima more than 8 mm apart. * p-value also significant for FWE correction, BA: Brodmann Area, MNI: Montréal Neurological Institute, R: right, L: left. (DOCX) [file pone.0198894.s002.docx]

**Supplementary Information**

Functional reorganization of the conceptual brain system after deafness in early childhood

Natalie M. Trumpp and Markus Kiefer

Ulm University, Department of Psychiatry, Ulm, Germany

**S2 Table.** **Exclusive masking analyses.**

| **Brain region** | **BA** | **MNI coordinates (mm)** | **T** | **P_Voxel_** | **Cluster size** | **P_Cluster_** |
| --- | --- | --- | --- | --- | --- | --- |
| ***Activity to sign language in deaf subjects exclusively masked with activity to action observation in deaf subjects*** | | | | | | |
| Superior temporal R | 42 | 64 -34 16 | 20.62 | < 0.0001* | 975 | < 0.0001* |
| Middle temporal R | 21 | 56 -22 -8 | 13.51 | < 0.0001* |  |  |
| Middle temporal R | 21 | 54 4 -18 | 13.18 | < 0.0001* |  |  |
| Middle occipital R | 17 | 24 -98 6 | 19.23 | < 0.0001* | 4616 | < 0.0001* |
| Calcarine L | 17 | 2 -86 -2 | 17.32 | < 0.0001* |  |  |
| Superior occipital L | 17 | -12 -104 12 | 17.14 | < 0.0001* |  |  |
| Inferior frontal pars opercularis L | 44 | -48 12 28 | 17.30 | < 0.0001* | 982 | < 0.0001* |
| Inferior frontal pars triangularis L | 45 | -52 36 12 | 12.75 | < 0.0001* |  |  |
| Inferior frontal pars triangularis R | 45 | 52 42 18 | 15.27 | < 0.0001* | 695 | < 0.0001* |
| Inferior frontal pars triangularis R | 48 | 54 18 22 | 12.00 | < 0.0001* |  |  |
| Inferior frontal pars triangularis R | 48 | 50 22 16 | 11.06 | < 0.0001* |  |  |
| Superior parietal R | 7 | 34 -62 58 | 14.99 | < 0.0001* | 169 | < 0.0001* |
| Inferior parietal R | 40 | 40 -48 50 | 9.71 | < 0.0001* |  |  |
| Hippocampus R | 20 | 22 -26 -2 | 14.29 | < 0.0001* | 333 | < 0.0001* |
| Hippocampus R | 20 | 28 -14 -10 | 8.53 | < 0.0001* |  |  |
| Supplementary motor area L | 6 | 0 10 60 | 13.38 | < 0.0001* | 398 | < 0.0001* |
| Supplementary motor area L | 6 | -6 2 66 | 9.10 | < 0.0001* |  |  |
| Supplementary motor area L | 8 | -4 24 52 | 8.31 | < 0.0001* |  |  |
| Middle temporal L | 21 | -50 0 -20 | 13.04 | < 0.0001* | 1117 | < 0.0001* |
| Middle temporal L | 20 | -58 -24 -12 | 12.55 | < 0.0001* |  |  |
| Middle temporal L | 22 | -58 -4 -12 | 12.28 | < 0.0001* |  |  |
| Hippocampus L | 20 | -20 -26 -6 | 12.98 | < 0.0001* | 244 | < 0.0001* |
| Precentral L | 6 | -46 0 56 | 11.79 | < 0.0001* | 130 | < 0.0001* |
| Inferior orbito-frontal R | 47 | 34 26 -8 | 10.72 | < 0.0001* | 107 | < 0.0001* |
| Middle frontal R | 6 | 44 0 60 | 10.58 | < 0.0001* | 234 | < 0.0001* |
| Middle frontal R | 6 | 50 6 54 | 10.39 | < 0.0001* |  |  |
| Precentral R | 6 | 48 8 42 | 9.55 | < 0.0001* |  |  |
| Insula L | 47 | -32 24 -2 | 9.92 | < 0.0001* | 172 | < 0.0001* |
| Inferior orbito-frontal L | 47 | -40 26 -6 | 9.52 | < 0.0001* |  |  |
| Inferior orbito-frontal L | 46 | -48 46 -2 | 9.78 | < 0.0001* | 66 | < 0.0001* |
| Inferior orbito-frontal L | 47 | -50 38 -4 | 8.79 | < 0.0001* |  |  |
| Inferior parietal L | 40 | -40 -46 54 | 9.68 | < 0.0001* | 78 | < 0.0001* |
| Hippocampus R | 35 | 16 -6 -14 | 9.21 | < 0.0001* | 20 | < 0.0001* |
| Amygdala R | 34 | 22 0 -18 | 8.20 | < 0.0001* |  |  |
| ***Contrast deaf vs. hearing in the animacy decision task exclusively masked with activity to sign language in deaf subjects*** | | | | | | |
| Middle temporal L | 21 | -44 -18 -8 | 7.14 | < 0.0001* | 1012 | < 0.0001* |
| Superior temporal L | 22 | -50 -6 -8 | 5.93 | < 0.0001 |  |  |
| Superior temporal L | 42 | -55 -30 12 | 5.67 | < 0.0001 |  |  |
| Superior temporal R | 22 | 54 -32 8 | 6.29 | < 0.0001* | 1137 | < 0.0001* |
| Superior temporal R | 22 | 50 -26 4 | 6.23 | < 0.0001* |  |  |
| Superior temporal R | 22 | 62 -20 4 | 6.20 | < 0.0001* |  |  |
| Inferior frontal pars opercularis R | 44 | 54 10 28 | 4.83 | < 0.0001 | 49 | 0.046 |
| Lingual R | 18 | 14 -66 -4 | 4.68 | < 0.0001 | 220 | < 0.0001* |
| Vermis |  | 4 -60 -4 | 4.21 | < 0.0001 |  |  |
| Calcarine L | 18 | -10 -70 22 | 4.63 | < 0.0001 | 723 | < 0.0001* |
| Cuneus L | 18 | 6 -72 22 | 4.56 | < 0.0001 |  |  |
| Calcarine R | 18 | 24 -62 16 | 4.54 | < 0.0001 |  |  |
| Lingual L | 19 | -18 -46 0 | 4.56 | < 0.0001 | 85 | 0.012 |
| Postcentral R | 3 | 52 -4 32 | 4.37 | < 0.0001 | 53 | 0.039 |
| Precentral R | 6 | 42 -8 40 | 3.84 | < 0.0001 |  |  |
| Lingual L | 18 | -10 -72 0 | 4.22 | < 0.0001 | 88 | 0.010 |
| Lingual L | 18 | -16 -66 -6 | 4.18 | < 0.0001 |  |  |
| Cerebelum L |  | -8 -58 -6 | 3.59 | 0.0001 |  |  |
| Precentral R | 4 | 18 -28 56 | 4.14 | < 0.0001 | 57 | 0.033 |

Shown are peak voxels with highest t-values for significant clusters and their local maxima more than 8 mm apart. ^*^ p-value also significant for FWE correction, BA: Brodmann Area, MNI: Montréal Neurological Institute, R: right, L: left.
